# PROBLEM SOLVING SKILLS AND CANCER SCREENING BEHAVIORS IN MIDDLE-AGED AND OLDER MEN IN THE U.S.

**DOI:** 10.1093/geroni/igz038.1204

**Published:** 2019-11-08

**Authors:** Shalini Sahoo, Roberto J Millar, Takashi Yamashita, Phyllis Cummins

**Affiliations:** 1 University of Maryland, Baltimore County, Baltimore, Maryland, United States; 2 Scripps Gerontology Center, Miami University, Oxford, Ohio, United States

## Abstract

Routine cancer screening is widely recognized as an effective strategy for reducing cancer mortality – the second leading cause of death in the U.S. Research shows cancer screening rates need to be improved, and men are less likely to uptake recommended screening than women. Cancer screening requires an array of tasks such as seeking up-to-date guidelines, making appointments, planning a hospital visit, and communicating with health care professionals in the complex health care systems. Importantly, modern health care systems are rapidly adopting technology such as web-based applications for information dissemination and communication with patients. This current study is designed to better understand the roles of problem-solving skills in the technology-rich environment (PSTRE) in two selected cancer screening behaviors among middle-aged and older men. We obtained nationally representative data with a sophisticated PSTRE assessment from the 2012/2014 Program for the International Assessment of Adult Competencies (PIAAC). Binary logistic regression models with survey weights were used to estimate the association between PSTRE scores (1 – 500 points) and two cancer screening behaviors of men who meet the recommended guideline of age between 45 to 74 years old (n = 1,168). Results showed that greater PSTRE scores were positively associated with prostate cancer screening (OR = 1.005, p < 0.05). Improvement in PSTRE may promote the specific cancer screening behaviors. Our findings also inform future interventions that seek to improve cancer screening among a vulnerable section of older populations.

